# Daily corticosterone rhythm modulates pineal function through NFκB-related gene transcriptional program

**DOI:** 10.1038/s41598-017-02286-y

**Published:** 2017-05-18

**Authors:** Sanseray da Silveira Cruz-Machado, Eduardo K. Tamura, Claudia E. Carvalho-Sousa, Vanderlei Amadeu Rocha, Luciana Pinato, Pedro A. C. Fernandes, Regina P. Markus

**Affiliations:** 1 Laboratory of Chronopharmacology, Department of Physiology, Institute of Biosciences, University of São Paulo (USP), 05508-090 São Paulo, SP Brazil; 2 Department of Biological Sciences, University of Santa Cruz (UESC), 45662-900 Ilhéus, BA Brazil; 3 São Paulo State University (UNESP), 17525-900 Marilia, SP Brazil

## Abstract

Melatonin and glucocorticoids are key hormones in determining daily rhythmicity and modulating defense responses. In nocturnal animals, corticosterone peaks at light/dark transition,while melatonin peaks at the middle of the night in both nocturnal and diurnal animals. The crosstalk between adrenal and pineal glands under inflammatory conditions indicates that corticosterone potentiates nocturnal melatonin synthesis by reducing the activity of NFκB. This transcription factor, which modulates the expression of a key enzyme in melatonin synthesis, is sharply reduced at the entrance of darkness in the rat pineal gland. In this study, we established the basis for understanding the crosstalk between adrenal and pineal glands in physiological conditions. Here we show that the expression of 70 out of 84 genes implied in defense responses exhibit a sharp reduction exactly at the entrance of darkness. Mifepristone impair the changes of 13 out of 84 genes, suggesting that the rhythm of corticosterone modulates pineal phenotype, as mifepristone also reduces the expression of *Aanat* and the nocturnal synthesis of melatonin. Therefore, darkness-induced synthesis of the pineal hormone, besides being controlled by the central clock located in the hypothalamus, is also influencedby glucocorticoids through the regulation of NFκB transcriptional program.

## Introduction

The daily temporal organization in mammals relies on the activity of two major endocrine glands: pineal and adrenal. The first transduces darkness, while the second signals the organism for the onset of activity^1, 2^. Rodents displaying nocturnal activity have both hormones produced in phase.

The endogenous master clock located in the suprachiasmatic nucleus (SCN) of the hypothalamus provides the timing for nocturnal melatonin synthesis by the pineal gland^3, 4^. The multisynaptic pathway links the SCN to the intermediolateral column of the spine, the superior cervical ganglion and the sympathetic post-synaptic neurons, which releases noradrenaline and activate pinealocytes β-adrenoceptors. Melatonin (N-acetyl-5-methoxytryptamine) synthesis involves the acetylation of 5-hydroxytriptamine (serotonin) by the enzyme arylalkylamine N-acetyltransferase (AANAT) and the methylation of N-acetylserotonin (NAS) by acetyl serotonin O-methyltransferase (ASMT). AANAT transcription is regulated by darkness^1^. In rats, the noradrenergic sympathetic input increases in 70–100 times the transcription of the gene and the activity of the enzyme.

The SCN also entrains the release of corticosterone by the adrenal glands at the light/dark transition, which is responsible for arousal^5^. Darkness-induced sympathetic output promotes the release of adrenocorticotropic hormone (ACTH) by the pituitary gland and the modulation of ACTH receptors located in the adrenal cortex^6^. Otherwise, inflammatory responses increase ACTH and corticosterone plasma levels in a non-rhythmic manner^7^.

The interplay between the two hormones has been evaluated in stressful conditions or in models of infection^8–11^. The anti-inflammatory effect of glucocorticoids mediated by activation of transcription programs is triggered by the nuclear translocation of the glucocorticoid receptor (GR)^12^. As for melatonin, its activity reduces oxidative stress and inhibits the nuclear translocation of the nuclear factor kappa B (NFκB), a pivotal transcription factor for inflammatory responses^13–15^. In stressful conditions, signalized by simultaneous activation of both α and β adrenoceptors, corticosterone reduces the synthesis of melatonin in the pineal gland^11^. On the other hand, this synthesis is stimulated by low stressful conditions when only β-adrenoceptors are triggered^8, 11^. Moreover, in healthy rats, trans-pineal perfusion of corticosterone, both at the light or dark phase leads to a significant increase in nocturnal melatonin output, with no change in daytime levels^9^, suggesting a putative interaction between the daily rhythms of corticosterone and melatonin.

The mechanism of action of corticosterone involves the suppression of the nuclear translocation of NFκB^16^, which binds to κB elements in the *Aanat* promoter, reducing the expression of this key enzyme in melatonin synthesis^17, 18^. We have previously shown that the constitutive expression of the NFκB homodimer p50/p50 increases along the light phase^19^. Entrance of darkness abruptly decreases NFκB nuclear content; otherwise, if lights are maintained ON no reduction is observed^19, 20^. Considering that NFκB regulates *Aanat* transcription and melatonin synthesis, both in health and pathophysiological conditions^20, 21^, and its sharp reduction occurs at the day/night transition, we evaluated whether the entrance of darkness also interfere in the expression of pivotal genes linked to inflammatory response. Our data show a new rhythmic pattern of gene expression in the rat pineal gland. At the light/dark transition the expression of 70 out of 84 genes involved in defense signaling pathways present a sharp alteration at the light/dark transition, indicating that the pineal gland perceives the moment of lights off. In addition, we show that the peak of corticosterone, linked to arousal, regulates pineal gland nocturnal melatonin synthesis.

## Results

### Glucocorticoid Receptor regulates TLR4/NFκB-related genes and melatonin synthesis in the rat pineal gland

**Figure 1:**
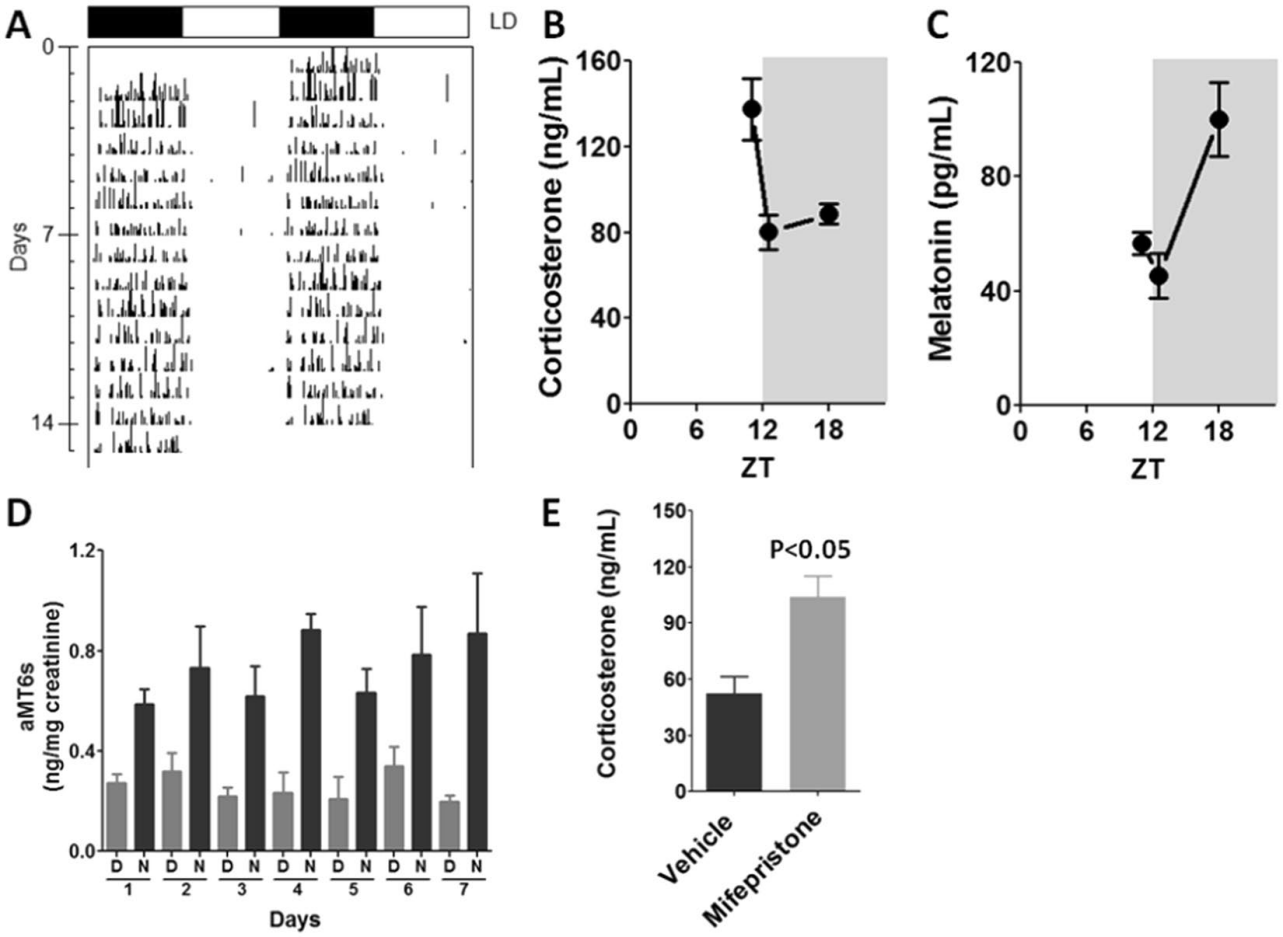
Daily rhythms in animals kept to a 12/12 h light/dark cycle. (**A**) Representative actogram of daily activity rhythm measured by means of water intake along 14 days. (**B**) Plasma levels of corticosterone at light/dark transition (ZT12L/ZT12D) and middle of the night (ZT18). (**C**) Plasma levels of melatonin at light/dark transition (ZT12L/ZT12D) and middle of the night (ZT18). (**D**) Daily rhythm of urinary excretion of 6-sulfatoxymelatonin (aMT6s) measured along 7 consecutive days during the day (**D**) and the night (N). (**E**) Effects of Mifepristone, a competitive antagonist of GR, in the plasma corticosterone levels measures at ZT18. The graphs show the mean ± SEM for 3–5 plasma hormone determinations or 4 urinary samples. Differences between groups were tested by Student’s t test.

Synchronization of the animals to a light/dark cycle (12/12 h) was confirmed by registering daily water intake rhythm (Fig. 1A), and plasma corticosterone and melatonin rhythms at the light/dark transition and middle of the night (Fig. 1B and C, respectively), as well as the profile of 6-sulfatoxymelatonin (aMT6s) urinary excretion along 7 days (Fig. 1D). All the parameters indicate that the animals are under healthy conditions. Corticosterone peaks at the light/dark transition and melatonin and its metabolite aMT6s during nighttime. Mifepristone, a competitive antagonist of GR, impairs the reduction of plasma corticosterone observed at ZT18 (Fig. 1E), suggesting that the well-known negative feedback induced by hypothalamic GR activation is impaired.

**Figure 2:**
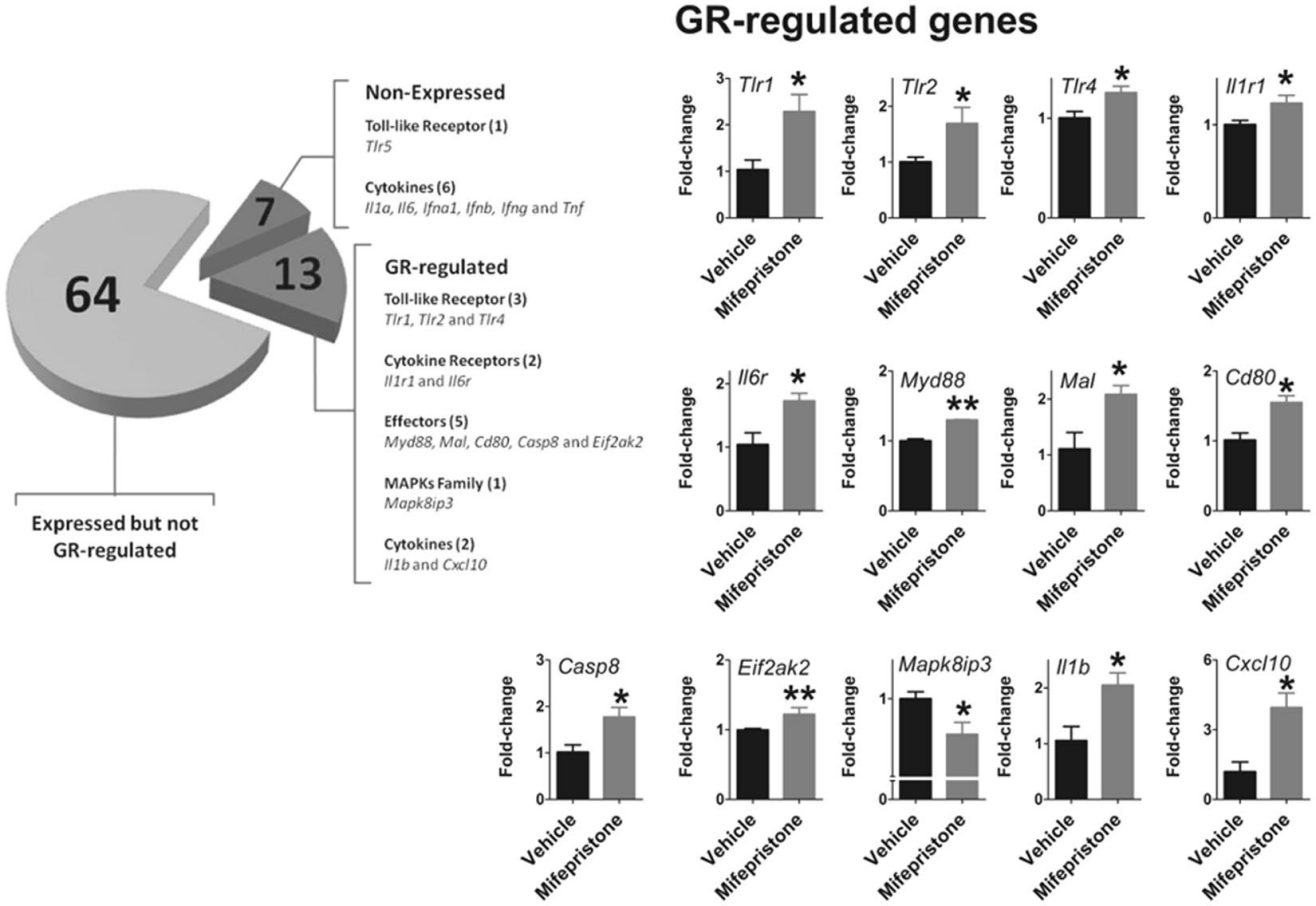
Corticosterone regulates the rhythmic transcriptional program related to NFκB signaling in rat pineal glands. Pineal glands from mifepristone-treated animals showed increased expression of *Tlr1*, *Tlr2*, *Tlr4*, *Myd88*, *Mal*, *Il1r1*, *Il6r*, *Il1b*, *Cd80*, *Casp8*, *Cxcl10*, *Eif2ak2* and decreased expression of *Mapk8ip3* gene at ZT12D. N = 3 glands per group. The graphs show the mean ± SEM. Means were compared by Student’s t test. *P < 0.05, **P < 0.01.

Given that melatonin synthesis is impaired by nuclear translocation of NFκB and corticosterone prevents this translocation^17, 20, 21^, we evaluated whether the increase in plasma corticosterone levels at observed ZT12 synchronizes the inhibition of gene programs regulated by NFκB in rats treated or not with mifepristone (Fig. 2). Thirteen out of 84 genes had their transcription altered by mifepristone (*Tlr1, Tlr2, Tlr4, Il1r1, Il6r, Myd88, Mal, Cd80, Casp8, Eif2ak2, Mapk8ip3, Il1b and Cxcl10*). Except for *Mapk8ip3*, whose expression was significantly lower than vehicle, all the other genes showed increased mRNA levels in pineal glands from mifepristone-treated rats (Fig. 2). As such, circulating corticosterone modulates the pineal gland phenotype at the rest/activity transition, which might have consequences for pineal gland functioning.

### TLR4-NFκB transcriptional program is rhythmically expressed in the rat pineal gland

In sequence, we evaluated the rhythmic expression of genes related to the NFκB transcriptional program. The expression of 84 genes related to the NFκB pathway was determined at five different time points: ZT0, ZT6, ZT12L, ZT12D and ZT18. To verify the relevance of the entrance of darkness, animals were killed just before (ZT12L) or after the lights off (ZT12D).

**Figure 3:**
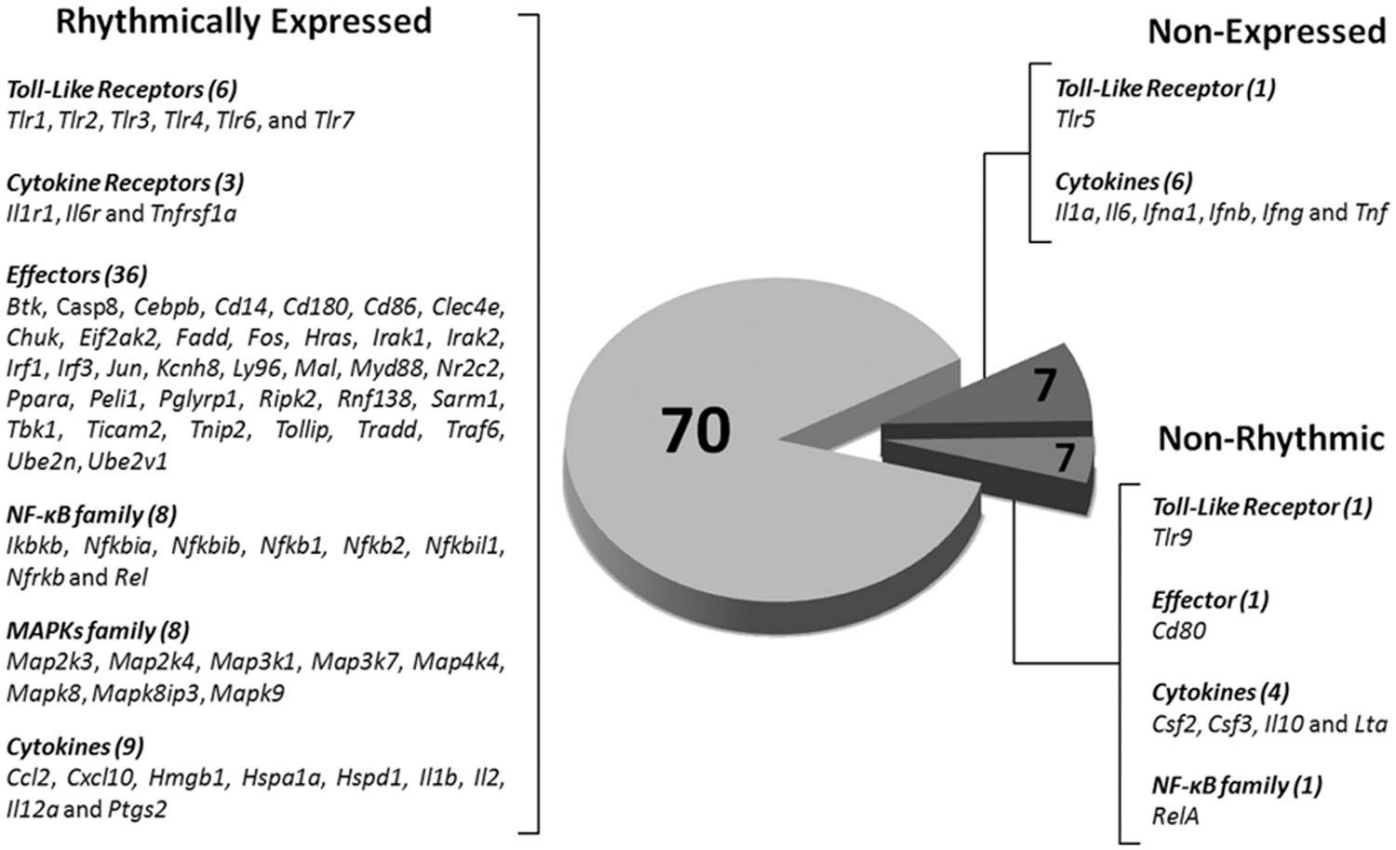
Constitutive rhythmic and non-rhythmic gene expression in pineal glands of rats euthanized at 5 different ZTs (0, 6, 12 L, 12D, 18) determined by qPCR array. Seven out of 84 genes were not expressed. Seventy genes showed rhythmic expression, while 7 genes present no rhythmic expression. Data were obtained from three pools of three pineal glands per time point.

**Figure 4:**
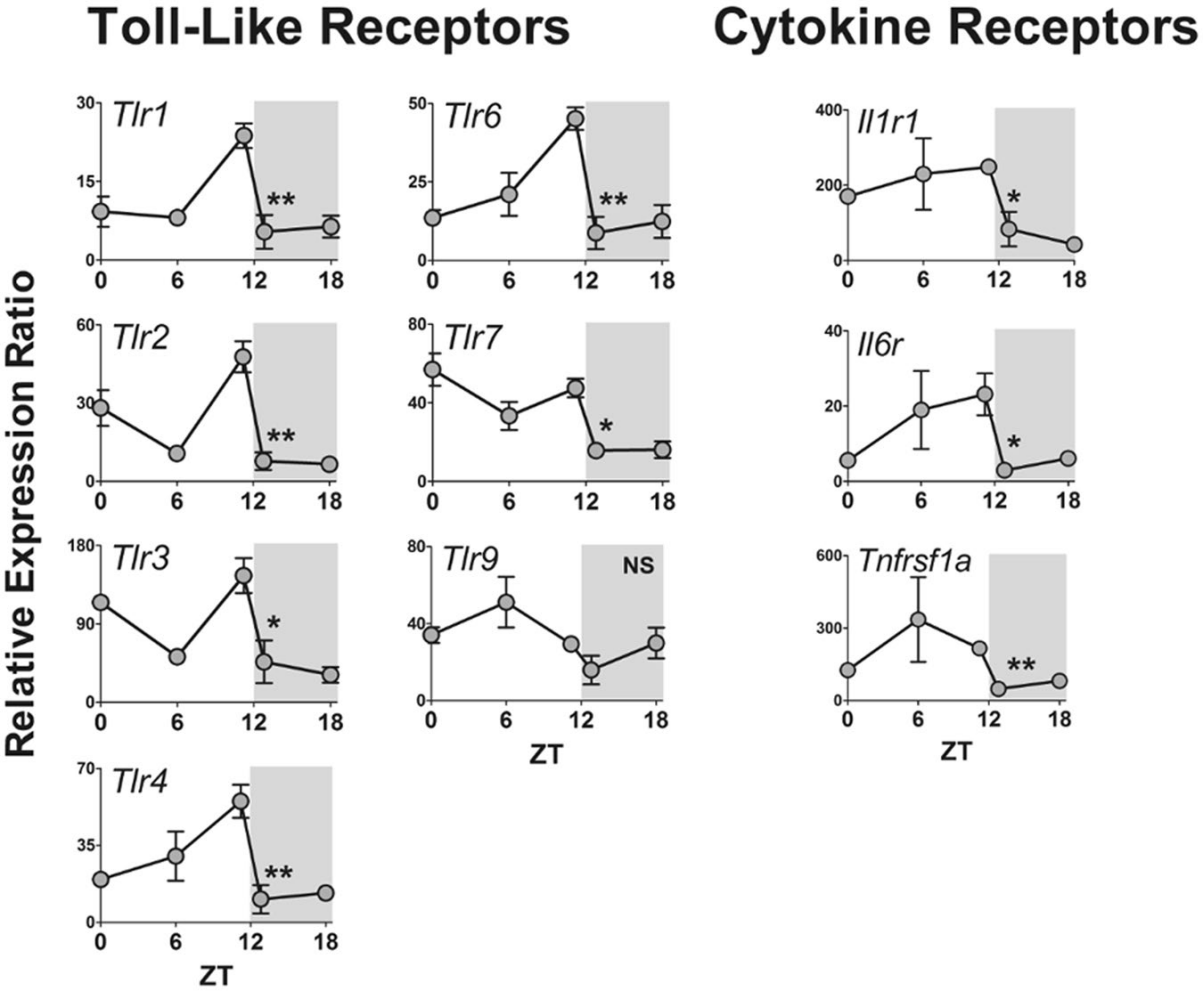
Daily pattern of gene expression coding for toll-like (TLR) and cytokine receptors in rat pineal glands. Gene expression was determined by qPCR. Values are shown as the relative expression ratio normalized by the reference gene *Rpl13*. Each value represents the mean ± SEM of three independent arrays per ZT. The difference in gene expression between ZT12L and ZT12D were compared by independent Student “t” test. *P < 0.05, **P < 0.01, NS = P > 0.05.

**Figure 5:**
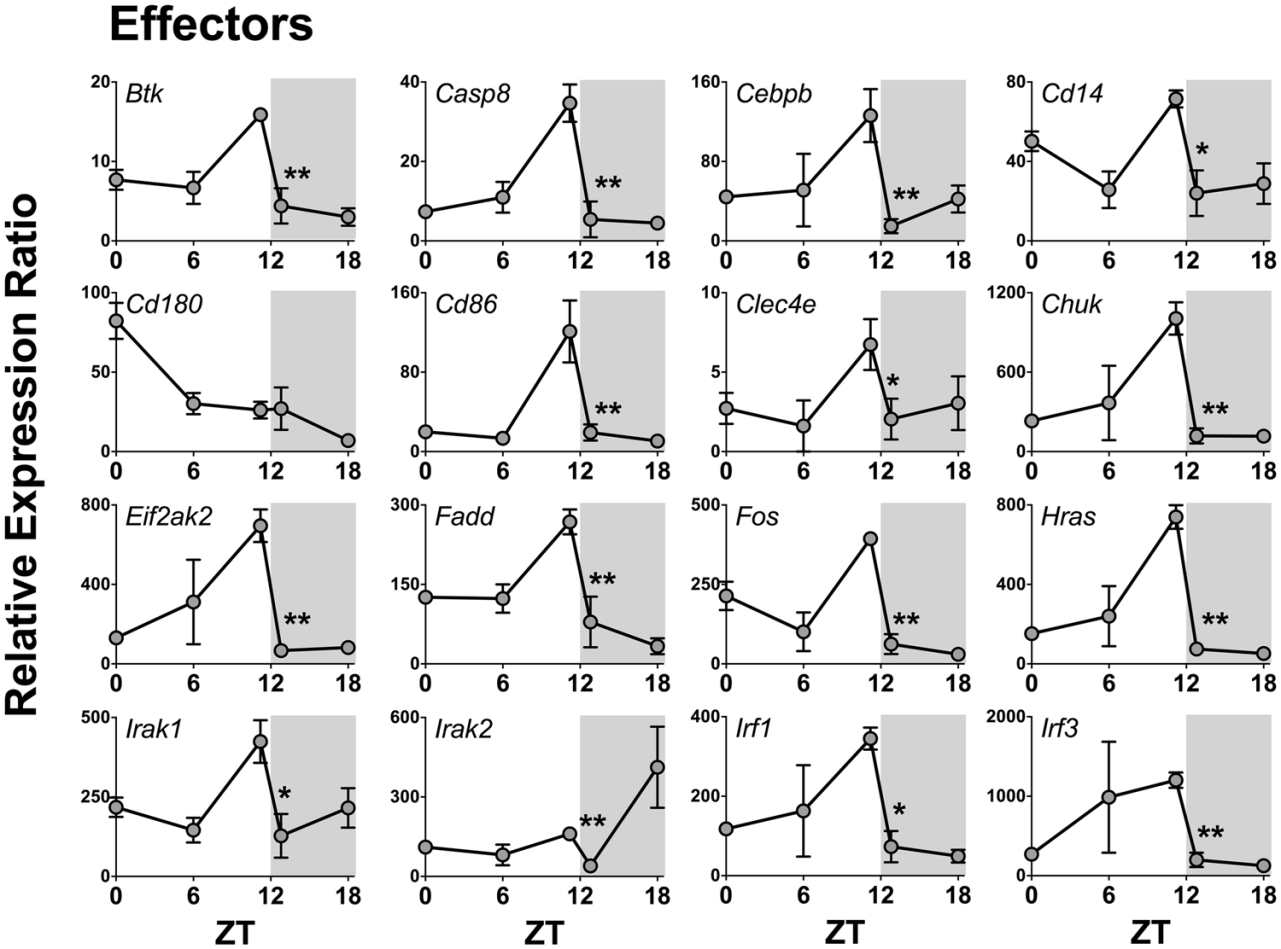
Daily pattern of gene expression coding for effectors of NFκB activation in rat pineal glands. Gene expression was determined by qPCR. Values are shown as the relative expression ratio normalized by the reference gene *Rpl13*. Each value represents the mean ± SEM of three independent arrays per ZT. The difference in gene expression between ZT12L and ZT12D were compared by independent Student “t” test. *P < 0.05, **P < 0.01, NS = P > 0.05.

**Figure 6:**
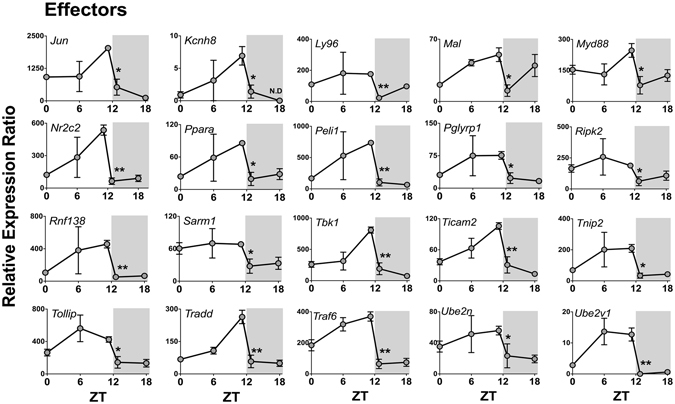
Daily pattern of gene expression coding for effectors of NFκB activation in rat pineal glands (continuation). Gene expression was determined by qPCR. Values are shown as the relative expression ratio normalized by the reference gene *Rpl13*. Each value represents the mean ± SEM of three independent arrays per ZT. The difference in gene expression between ZT12L and ZT12D were compared by independent Student “t” test. *P < 0.05, **P < 0.01.

**Figure 7:**
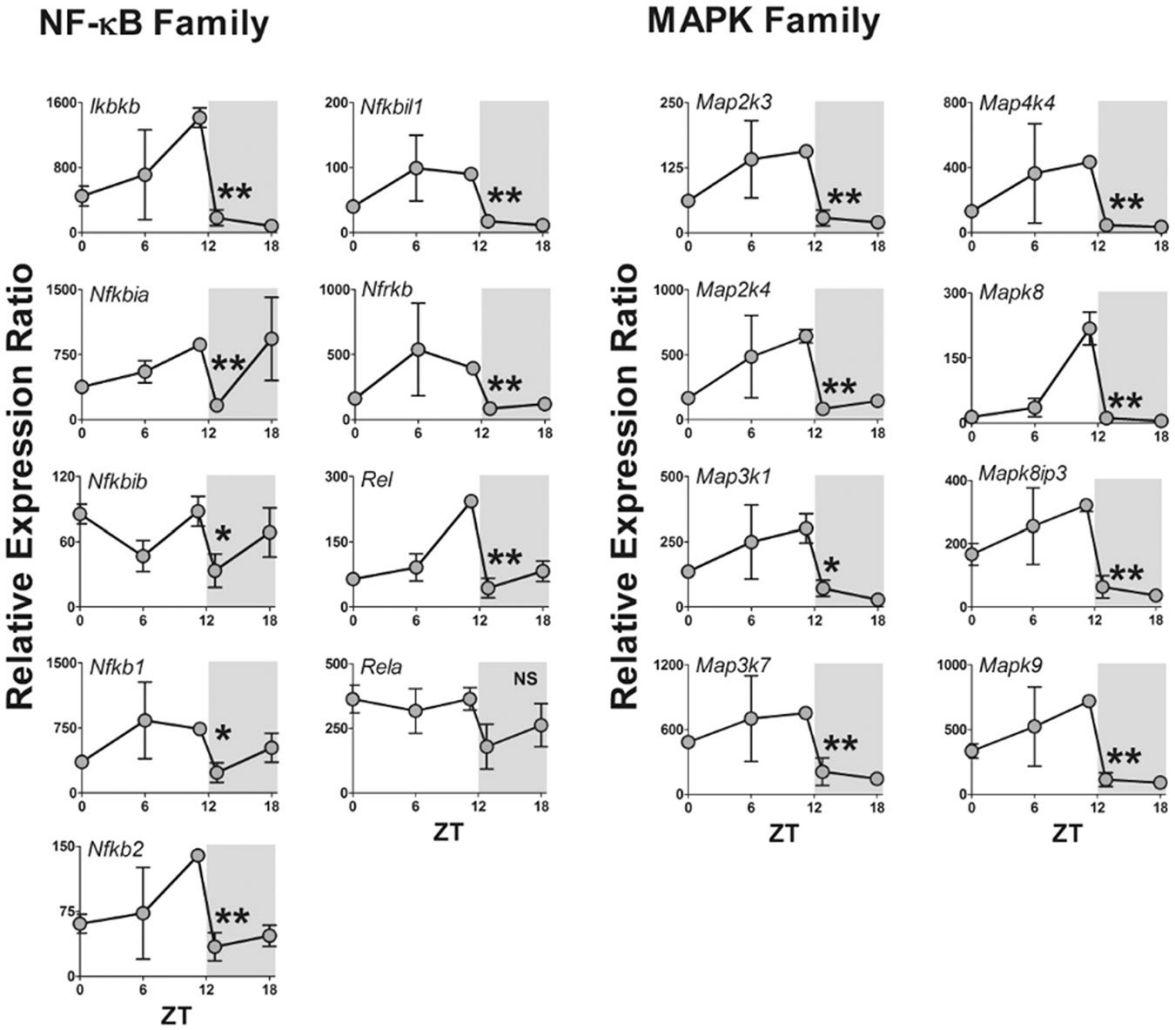
Daily pattern of gene expression coding for NFκB or MAPK families in rat pineal glands. Gene expression was determined by qPCR. Values are shown as the relative expression ratio normalized by the reference gene *Rpl13*. Each value represents the mean ± SEM of three independent arrays per ZT. The difference in gene expression between ZT12L and ZT12D were compared by independent Student “t” test. *P < 0.05, **P < 0.01, NS = P > 0.05.

**Figure 8:**
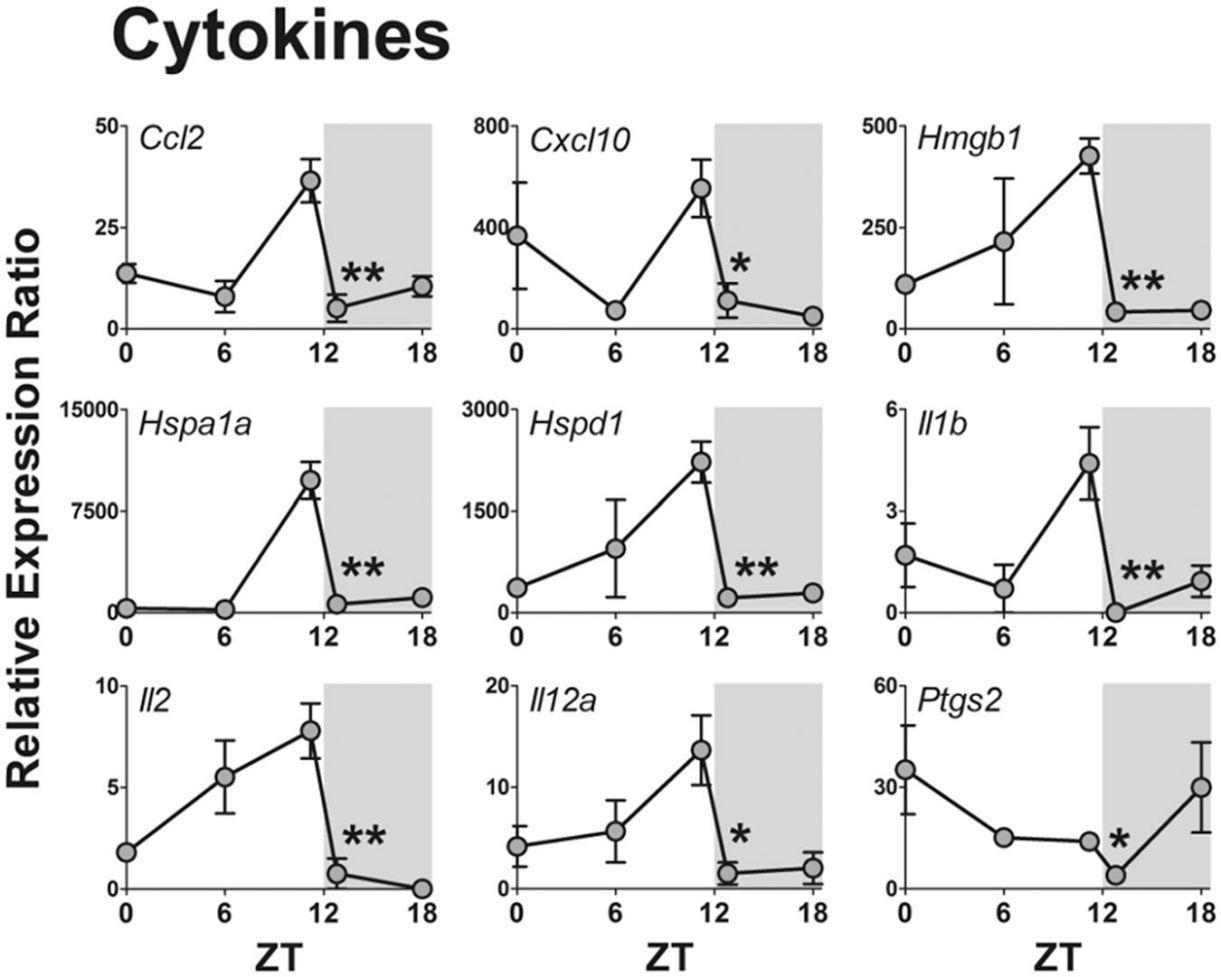
Daily pattern of gene expression coding for cytokines in rat pineal glands. Gene expression was determined by qPCR. Values are shown as the relative expression ratio normalized by the reference gene *Rpl13*. Each value represents the mean ± SEM of three independent arrays per ZT. The difference in gene expression between ZT12L and ZT12D were compared by independent Student “t” test. *P < 0.05, **P < 0.01.

Seven out of 84 genes were not expressed at any time (*Tlr5, Il1a*, *Il6*, *Ifna1*, *Ifnb*, *Ifng*, and *Tnf*), while seven genes were expressed in a non-rhythmic manner (*Tlr9, Cd80*, *Csf2*, *Csf3*, *Il10*, *Lta*, and *Rela*). The other 70 genes showed a robust daily rhythm: 69 presented a significant reduction at the light/dark transition and only one gene (*Cd180*) presented maximal expression at the dark/light transition (Fig. 3). Figures are presented according to functional groups: toll-like and cytokine receptors (Fig. 4), effectors (Figs 5 and 6), NFκB and mitogen-activated protein kinase (MAPK) families (Fig. 7) and cytokines (Fig. 8). An inspection of Figs 4 to 8 clearly shows that at light-to-dark transition (between ZT12L and ZT12D) there is an overall rapid and stereotyped reduction in the expression of the analyzed genes. This decrease was consistently observed among different experiments and for different classes of genes.

Among the TLRs, the transcription of six out of eight coding genes showed a fast reduction between ZT12L and ZT12D. *Tlr5* was not expressed and *Tlr9* did not show a significant daily rhythm or any difference in its expression levels at the light/dark transition (Fig. 4). The genes that codify inflammatory cytokine receptors (*Il1r1*, *Il6r* and *Tnfrsf1a*) also followed the rhythmic pattern observed for most of the TLRs (Fig. 4).

The expression of 37 out of 38 genes that codes for TLRs effectors of signaling also presented an abrupt decrease between ZT12L and ZT12D, including: membrane and intracellular adaptors; signaling effectors, such as *Cd14* and *Ly96* membrane adaptors; as well as *Myd88*, *Mal* (that codes TIRAP), *Rnf138* (that codes TRIF), and *Ticam2* (that codes TRAM) (Figs 5 and 6). Consistently, TLRs activation converges on IκB kinases (IKK1 or IKK2, coded by *Chuk* or *Ikbkb* genes, respectively), which followed the same rhythmic transcription program of the upstream genes.

NFκB dimers are composed by homo- or heterodimers formed by the proteins RelA, RelB, c-Rel, p50 and p52. Such NFκB dimers are pivotal for coupling membrane-activated TLRs to transcriptional programs. Except for *RelA*, the expression of *Nfkb1* (which codes p50/p105), *Nfkb2 (*which codesp52/p100) and *Rel*(which codes c-Rel) followed the same rhythmic pattern described for the upstream components (Fig. 7), with their expression at ZT12 before lights off being significantly higher than that observed after lights off. The gene RelB was not available in the qPCR array used; however, we determined the presence of the protein in pinealocytes (Supplementary Fig. l). A similar daily profile was observed for the eight genes, which code for MAPKs (*Map2k3*, *Map2k4*, *Map3k1*, *Map3k7*, *Map4k4*, *Mapk8*, *Mapk8ip3* and *Mapk9*).

We also evaluated the expression of genes that code for inflammatory mediators. We found that 13 out of 19 cytokines are expressed in rat pineal glands, with 9 presenting a rhythmic expression (*Ccl2*, *Cxcl10*, *Hmgb1*, *Hspa1a*, *Hspd1*, *Il1b*, *Il12a*, *Il2* and *Ptgs2)* (Fig. 8). The expression of 4 genes did not vary throughout light or dark phase (*Csf2*, *Csf3*, *Il10* and *Lta)*, and 6 genes were not expressed (*Il1a*, *Il6*, *Ifna1*, *Ifnb*, *Ifng* and *Tnf*). Notably, all of these genes, whose mRNA could not be detected in pineal glands from healthy rats, code for pro-inflammatory cytokines, which are expressed in the pineal gland only when rats are challenged by pathogen- (PAMPs) or danger-associated molecular patterns (DAMPs)^11, 22–25^.

The expression of some genes did not follow the rhythmic pattern indicated above. For example, *Cd80* was not rhythmically expressed. This membrane protein is detectedin stimulated antigen-presenting cells and in glial cells, being part of the co-stimulation of lymphocytes^26^. Although *Irak2* presented a daily rhythm, its expression was maximal at ZT18, in spite of a small but significant reduction between ZT12L and ZT12D. In turn, *Cd180* expression was not affected by the light/dark transition with its peak occurring at ZT0, which is the beginning of the light phase. In order to confirm that the expression of the detected genes is translated into protein expression, immunofluorescence in pinealocytes was carried out. Besides the proteins TLR4, TNFR1 and CD14, which was previously detected^22–24^ here we show that pinealocytes express IKK1, IKK2, IRAK, TIRAP, TRAF6, TRAF1, RelA, RelB, c-Rel, p51, p100, p105, IκBα, BCL3, caspase 8 and MEK7 (Supplementary Fig. 1). Therefore, the family of proteins related to this important signaling pathway is constitutively expressed and rhythmically regulated in pinealocytes. Another interesting observation in our immunofluorescence detection is that the subunits c-Rel, p52, RelA, RelB, p100 and p105 are restricted inactivated in the cytoplasm, as expected in cells obtained from non-stressed or non-inflamed animals.

We also determined whether TLR4 protein is expressed in rat pineal glands from animals killed at light (ZT2 and ZT10) or dark phase (ZT14, ZT18, ZT22). Here we detected a positive expression of TLR4 in the rat pineal gland. Considering the intensity of expression, we found a stronger immunostaining for TLR4 in glands from animals killed at ZT10 compared to ZT2. At dark phase, there is a reduction in the expression of TLR4 at ZT14, ZT18, and ZT22 compared to ZT10 (Supplementary Fig. 2). In summary, at the light/dark transition, parallel with the plasma corticosterone peak, we observed a synchronized reduction of the expression of genes related to the NFκB pathway.

**Figure 9:**
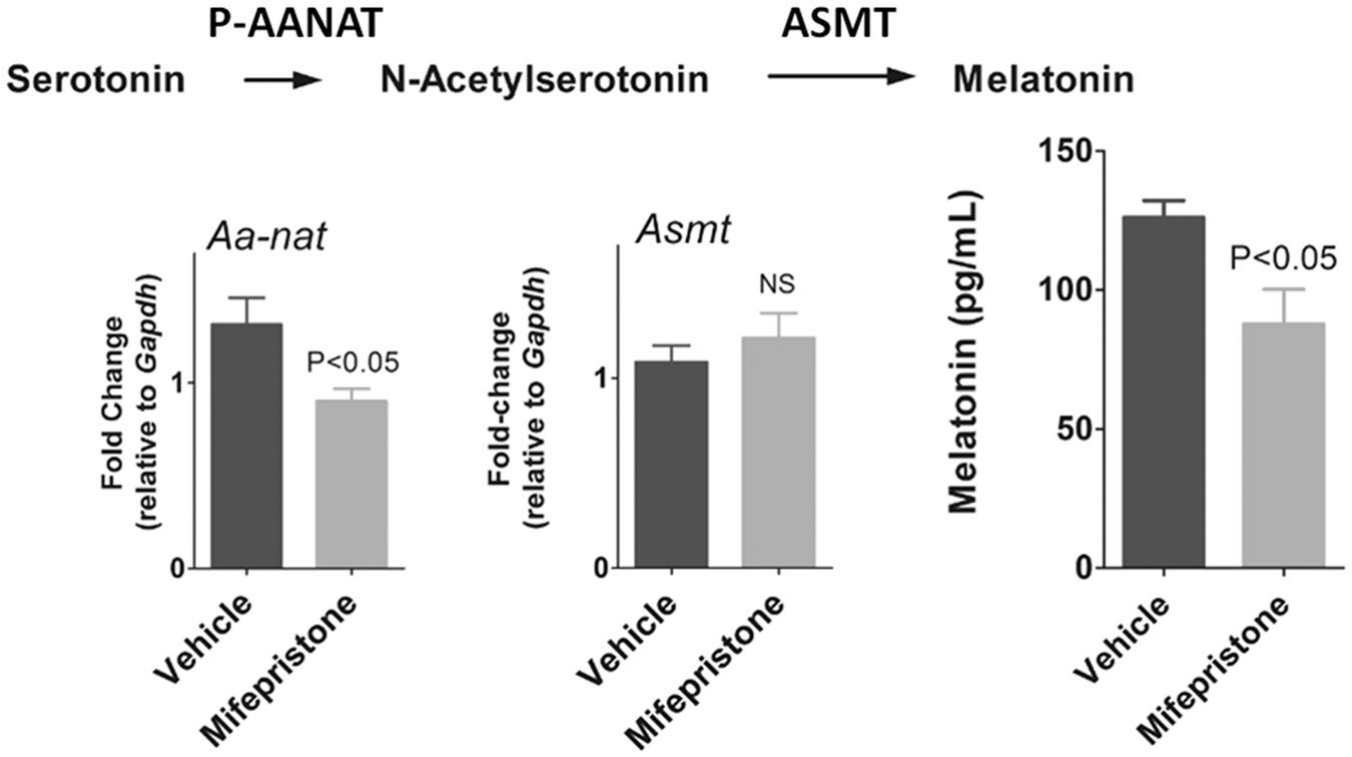
Effects of GR blockade on nocturnal expression of AA-NAT and ASMT coding genes and plasma levels of melatonin of rats killed at ZT18. Mifepristone-treated animals showed lower circulating melatonin levels and reduced pineal expression of *Aanat* levels at ZT18. N = 5–8 animals per group. The graphs show the mean ± SEM. Means were compared by Student’s t test.

Given that NFκB activation reduces the expression of *Aanat*, impairing the synthesis of melatonin, we evaluated whether the corticosterone peak could be responsible for allowing a proper increase in *Aanat* expression at the dark phase of the day. This question was approached by inhibiting glucocorticoid receptors with mifepristone and evaluating the expression of *Aanat* and *Asmt* in the pineal gland and melatonin plasma concentration at ZT18, i.e., in the middle of the dark phase. Indeed, mifepristone reduced the relative expression of *Aanat* and the plasma concentration of melatonin (Fig. 9). Inhibiting glucocorticoid receptors did not modify the expression of *Asmt*.

## Discussion

Adrenal corticosterone and pineal melatonin, two hormones directly implicated in the temporal organization of daily rhythms, transduce rest/activity and light/dark rhythms, respectively^1, 2^. Corticosterone daily rhythm is not in phase with melatonin rhythm in diurnal animals, while in nocturnal animals the rhythms are in phase. The interaction of glucocorticoids and melatonin during stressful^8, 10^ and inflammatory conditions^11^ are well addressed. Otherwise, the physiological interaction between adrenal and pineal gland is poorly understood.

Pineal gland is a target for glucocorticoids as it expresses glucocorticoid receptors^16^, and corticosterone has a dual effect on melatonin synthesis^11^. Mild stress^8^ or corticosterone infusion in rats^9^ increases nocturnal β-adrenoceptors-induced melatonin synthesis. On the other hand, when both α- and β-adrenoceptors are activated due to stressful conditions, corticosterone reduces nocturnal melatonin output^11^. The relationship between adrenal and pineal gland in non-stressful conditions is still an open question. Previous data from our group showed that the nuclear content of the NFκB homodimer p50/p50 in the rat pineal gland is sharply reduced at the entrance of darkness or subjective night^19^. This reduction of nuclear NFκB was not mediated by sympathetic activation, as it was not blocked by propranolol^19^. Considering that the same effect is observed in animals synchronized to environmental lighting or in free-running conditions^19^, we hypothesized that it follows the rest/activity cycle, being therefore, a good model for exploring physiological interaction between adrenal and pineal glands.

Here we show that in physiological conditions a package of seventy genes related to defense response is synchronized by the light/dark transition in rat pineal gland, and follows a similar pattern observed to nuclear translocation of p50/p50 NFκB protein: a cumulative diurnal increase peaking at ZT12L, followed by a rapid decrease at ZT12D. It is noteworthy that genes coding for receptors (*Tlr2* and *Tlr4*) and their ligands (*Hmgb1*, *Hsp60* and *Hsp70*) follow the same profile. A daily rhythm of pineal genes was previously evaluated during day and nighttime in rats maintained at 14 h/10 h light/dark regimen^27, 28^. However, this study did not evaluate the light/dark transition. Thus here we show that the entrance of darkness is an important time point for controlling pineal function.

In nocturnal animals, light/dark transition coincides with corticosterone peak responsible for preparing the animals for activity. Taking into account that this peak sharply reduces the expression of genes known to impair *Aanat* transcription^9, 19, 22, 23^, we raised the hypothesis that endogenous corticosterone could also modulate the amplitude of melatonin peak. Our data confirmed this hypothesis, as the blockade of glucocorticoid receptors reduced the transcription of *Aanat* and nocturnal circulating melatonin levels. It is noteworthy that among the genes modulated by glucocorticoid are those that codifies for *Tlr1*, *Tlr2*, *Tlr4* and for pro-inflammatory cytokine receptors (*Il1r1* and *Il6r*). Activation of these receptors has been shown to block sympathetic-induced synthesis of melatonin^22–24^. Therefore, GR activation at the light/dark transition facilitates the pineal gland to fully respond to sympathetic transduction of environmental darkness and also increases the ability of the gland to detect PAMPs, DAMPs and pro-inflammatory cytokines. As rats are nocturnal animals, we need to speculate about diurnal animals. In this case, corticosterone peak occurs at the entrance of daytime; therefore, *Aanat* transcription should be also high during daytime. Indeed, this is the case, as sympathetic input in diurnal animals only controls the enzyme activity but not the transcription of the gene^1^. Indeed, AA-NAT degradation by proteasome proteolysis occurs just after its synthesis. Sympathetic input protects AA-NAT from entrance in the proteasome^29^. As propranolol did not block the sharp reduction in NFκB nuclear translocation^19^ and endogenous corticosterone regulates only 13 out of 70 genes altered at the light/dark transition, other inputs to pineal gland for translating darkness should be considered. Pineal gland perceives environmental lighting through the well-studied sympathetic input^1, 2, 29, 30^ and through direct central projection from a thalamic structure involved in vertebrate circadian organization (intergeniculate leaflet, IGL)^31^. IGL receives information from the retina, and directly projects to the pineal gland probably making synaptic contacts with pinealocytes^32^. Indeed, IGL sectioning reduces the N-acetylserotonin synthesis^33^. Therefore, the sharp change in gene transcription at the light/dark transition could be the sum of different inputs reaching the pineal gland.

In summary, the data here obtained clearly show that the rat pineal gland is capable to perceive the entrance of corticosterone peak before darkness to regulate the nocturnal synthesis of melatonin. This is translated by a sharp reduction in the expression of genes classically linked to defense responses. Part of this effect is mediated by endogenous corticosterone-GR activity. Although sympathetic activity is the most well characterized signal driving the translation of darkness in the pineal gland^1, 3, 4, 9, 19, 27–30^, the present study provides new insights for a cooperative transcriptional program that adjusts the rat pineal gland to two cycles, namely the rest/activity and the light/dark cycles. In rats the two cycles are in phase, and our data strongly suggest that both are necessary for a full translation of the environmental darkness in driving pineal nocturnal synthesis of melatonin. As such, our data identifies that corticosterone rhythm is an important regulator of pineal function. Our data also provide a new molecular basis for a better understanding of the temporal organization and synchronization of the endocrine system.

## Methods

### Animals

Adult male Wistar rats (10-weeks-old, 300–320 g) were obtained from the animal facility of the Department of Physiology (IB-USP, São Paulo, Brazil), and kept under a 12/12 h light/dark cycle (LD, lights on at 07:00; *Zeitgeber* time 0 or ZT0). Animals received water and food *ad libitum*, and were killed by decapitation, at intervals of 6 hours (ZT0, ZT6, ZT12, ZT18). At the light/dark transition (ZT12) there were two groups: one killed 15 min before and the other 30 min after lights off (ZT12L, ZT12D). Procedures were approved by IB-USP Ethical Committee (CEUA license number 115/2010) and performed according to Brazilian law for scientific use of animals (Federal Law number 11.794/2008).

Daily rhythmic pattern of the animals was determined according to the daily water intake (activity)^34^, urinary excretion of 6-sulfatoxymelatonin (aMT6s), and plasma level of corticosterone and melatonin at light/dark transition and ZT18, respectively (Fig. 1).

### Drugs

Penicillin/streptomycin, HEPES, bovine albumin fraction V, dithiothreitol (DTT), glycerol, tris-HCl, glycine, glucose, PMSF, NP-40, paraformaldehyde, poli-L-lysine, mifepristone, magnesium chloride, sodium chloride, trypsin, and trypsin inhibitor were purchased from Sigma (St Louis, MO, USA); EDTA, sodium hydrogen carbonate, potassium dihydrogen phosphate, potassium chloride and disodium hydrogen phosphate were purchased from Merck (Rio de Janeiro, Brazil). Dulbecco’s modified eagle medium (DMEM), fetal bovine serum, TRIzol reagent, SuperScript III Reverse Transcriptase, Taq Polymerase, T4 polynucleotide kinase, and 6-diamidino-2-phenylindole (DAPI) were purchased from Life Technology (Calrsbad, CA, USA). Saponin and tween 20 were purchased from AMRESCO (Solon, OH, USA).

### *In vivo* antagonism of glucocorticoid receptors

Animals received two mifepristone injections (10 mg/kg, i.p.). The first was at the middle of the night (ZT18) before the experiment, and the second was 1 hour prior to lights off at the experimental day. The control group received vehicle (2 mg/mL carboxymethil cellulose solved in isotonic saline plus 20% tween 20, 5% ethanol, i.p.). Mifepristone efficacy was proved by the increase in circulating corticosterone due to the inhibition of the hypothalamic feedback (Fig. 1E).

### Corticosterone and melatonin measures

Plasma corticosterone (corticosterone RIA kit, ImmuChem, MP Biomedicals, NY, USA, detection limit: 5.0 nM) and melatonin (melatonin ELISA kit, IBL, Hamburg, Germany, detection limit: 3.0 pg/mL) were determined by radioimmunoassay, and enzyme-linked immunosorbent assay, respectively, according to manufacturer’s instruction.

### Gene transcription

RNA extraction and cDNA preparation was carried out as previously described^24^. Briefly, total RNA was extracted from each isolated pineal gland using TRIzol according to the manufacturer’s instruction. Complementary DNA (cDNA) was generated from 0.5 μg of total RNAusing random primers (65 °C, 5 min) and SuperScript III Reverse Transcriptase (200 U; 25 °C for 10 min, 50 °C for 55 min, 70 °C for 15 min).

The expression of 84 genes related to inflammatory signaling were investigated in rat pineal glands obtained at ZT12D, for experiments under GR antagonism by mifepristone, or at 5 time-points throughout the light/dark phase in naïve animals: ZT0, ZT6, ZT12L, ZT12D and ZT18. All the genes analyzed in the array kit are shown in the Supplementary Table 1 and Fig. 3. Gene transcription was evaluated by a commercial qPCR array kit (rat Toll-Like receptor signaling pathway, PARN-018A, SABioscience, Frederick, MD, USA) according to manufacturer’s instruction using the iCycler 5 (Bio-Rad, Hercules, CA, USA) thermocycler. This study was carried out using 9 different pineal glands per time point, whose cDNA were pooled to make three samples (3 glands per sample) and run in different plates. In addition to the 84 genes tested, each plate contained 5 housekeeping genes for quantitative analyses (*Rplp1, Rpl13a, Hprt1, Ldha* and *Actb)* and specific controls for genomic DNA contamination, RNA quality and efficiency (E). Only assays that passed in the internal controls were used for quantitative analysis (at least three assays per ZT). For data analysis, the CT expression of housekeeping genes was tested by GeNorm software in order to identify the most stable reference gene based on the geometric mean of expression. Most of the reference genes passed in the test, but for the relative expression ratio (R) analysis we chose the *Rpl13*, as it was the most stably expressed gene among samples. The relative gene expression is presented as the ratio between the cycle threshold of the gene of interest (GOI) and the cycle threshold of the reference gene (*Rpl13)* according to the formula provided by Pfaffl^35^. R = E_HKG_^(CTHKG)^/E_GOI_^(CTGOI)^; HKG = housekeeping; GOI = gene of interest.

Quantitative PCR was also carried out for the analysis of *Aanat*, *Asmt* and *Gapdh* gene expression based on sequences obtained from GenBank. All primers exhibited efficiency between 90% to 107%. The qPCR amplification was performed using cDNA (1 μL) in a final volume of 25 μL containing 2X SYBR Green mastermix (Invitrogen) supplemented to final concentrations of 1.5 mM MgCl_2_, 0.4 mM dNTPs, 2 units Taq polymerase, and each sense- and antisense-specific primers for *Aanat* (Forward: 5′-AGCGCGAAGCCTTTATCTCA-3′ and Reverse: 5′-AAGTGCCGGATCTCATCCAA-3′), *Asmt* (Forward: 5′-AGCGCCTGCTGTTCATGAG-3′ and Reverse: 5′-GGAAGCGTGAGAGGTCAAAGG -3′XXX) or *Gapdh* (Forward: 5′-TTCTTGTGCAGTGCCAGCC-3′ and Reverse: 5′-GTAACCAGGCGTCCGATACG-3′) at 50 nM final concentration. Each experimental cDNA was run in triplicates in 96-well plates. The assays were performed using i5 thermocycler (Bio-Rad, Hercules, CA, USA) in the following conditions: 2 min at 55 °C, 10 min at 95 °C, followed by 40 cycles of 15 seconds at 95 °C and 60 seconds at 60 °C then 81 cycles of 10 seconds at 55° and melting analysis. *Gapdh* expression, which did not vary along time under our experimental conditions, was chosen as a reference gene in all gene expression reactions and used to quantify the fold-change by the delta-delta CT method.

### Pinealocyte culture

Rat pinealocytes were isolated accordingly to da Silveira Cruz-Machado *et al*.^22^. Briefly, pineal glands were removed from animals killed at ZT10 and placed in cold phosphate-buffered saline (PBS, 0.25 M sodium chloride, 0.01 M potassium chloride, 0.04 M disodium hydrogen phosphate, 0.004 M sodium dihydrogen phosphate). Pinealocytes were obtained by trypsin digestion (0.25%, 37 °C, 15 min) followed by mechanical dispersion in the presence of trypsin inhibitor (0.3%) in buffer solution containing (mM): NaCl 120, KCl 5, NaHCO_3_ 25, KH_2_PO_4_ 1.2, glucose 12, and 0.1% w/v bovine serum albumin. After centrifugation (15 min, 1000 g) the cells were resuspended in DMEM supplemented with 10% v/w fetal bovine serum (heat-inactivated), 100 U/mL penicillin, and 100 μg/mL streptomycin (pH 7.4). The total number of cells and fractional survival was estimated by Trypan blue exclusion. The survival rate was 90% or higher. Cells (0.5 × 10^5^) were seeded on poly-L-lysine-coated 8-well chamber slide and maintained at 37 °C, 5% CO_2_ for 18 h prior to immunocytochemistry analyses.

### Immunocytochemistry

Pinealocytes were washed twice in PBS, fixed in 4% cold paraformaldehyde (10 min), and permeabilized with PBS supplemented with saponin 0.5% at room temperature. The non-specific binding sites were blocked by solution containing bovine serum albumin fraction V (BSA 1%), 3% normal serum and glycine 0.3 M for 60 min. The preparation was then incubated with primary antibody for 18 h at 4 °C (Supplementary Table 2) followed by the appropriate secondary antibody conjugated to the anti-rabbit (1:400, SC-2012; Santa Cruz Biotechnologies) or anti-mouse (1:200, M30201, Invitrogen) FITC for 1 h at room temperature. Nuclei were stained with 4′,6-diamidino-2-phenylindole (DAPI, 300 μM, 5 min) at room temperature. Antibodies were diluted in blocking buffer. Negative controls were performed by omitting primary antibodies from the procedure and its substitution for normal serum from the same species. Staining was completely abolished under these conditions. Cells were observed by confocal laser scanning microscopy Zeiss LSM 510 (Zeiss confocal software, Berlin, Germany) with a 40x oil-immersion objective. FITC and DAPI were excited at 488 nm (Argon laser) and 364 nm (enterprise laser), respectively, and the emitted fluorescence for FITC and DAPI was measured at 515–530 and 435–485 nm, respectively.

### Immunohistochemistry

Expression of TLR4 in the rat pineal cryosections was performed accordingly to da Silveira Cruz-Machado *et al*.^22^. Briefly, animals were deeply anesthetized by intramuscular injection of ketamine (160 mg/kg) plus xylazine (40 mg/kg) and perfused transcardially with 150 mL saline solution followed by 1000 mL of cold 4%paraformaldehyde fixative solution, pH 9.5. The perfusion was performed at two time-points at light phase (ZT2 and ZT10) or three different time points at dark phase under red light (ZT14, ZT18, ZT22). It is noteworthy to mention that in this experiment we have waited 2 hours after lights off because of differences between coding gene expression to protein translation. Each pineal gland was removed from the skull and cryoprotected in the same fixative solution plus 20% sucrose overnight, followed by 30% sucrose in PBS for 14 hr, embedded in Tissue Tek freezing medium, rapidly frozen in dry ice and stored at −80 °C. Cryostat sections (20 µm) were fixed for 30 min in freshly prepared 4%paraformaldehyde in PBS. Following 5 min of incubation with 0.1 M glycine, sections were incubated with blocking solution (3% albumin and 0.01% saponin in PBS) for 1 hr at room temperature. The Avidin-Biotin Blocking kit (Vector, SP2001, Burlingame, CA, USA) was used to block endogenous biotin. Rabbit polyclonal antibody against rat TLR4 (1:200; Abcam) was incubated overnight at 4 °C followed by incubation with appropriated secondary antibody conjugated to biotin (1:200; Abcam) for 1 hr at room temperature. The Avidin-Biotin-Complex (Vectastain Elite kit; Vector, PK-6105, Burlingame, CA, USA) staining system was used to localize biotinylated antibody. Peroxidase activity was revealed with 3, 3′-diaminobenzidine (DAB substrate kit for Peroxidase; Vector, SK-4100) according to manufacturer instruction. The sections were counterstained with methylene blue. Controls were performed by the omission of the primary antibodies from the procedure. Staining was completely abolished under these conditions.

### Data analysis

Data are presented as mean ± S.E.M. Statistical analysis was performed using the unpaired Student’s t test or ANOVA followed by Newman–Keuls post-test. Values of p < 0.05 were considered statistically significant.

## Electronic supplementary material

Supplementary information
