# Supplementary material for: Daily corticosterone rhythm modulates pineal function through NFκB-related gene transcriptional program
Source: Sci Rep. 2017 May 18;7:2091. doi: 10.1038/s41598-017-02286-y (PMC5437068; doi:10.1038/s41598-017-02286-y)
Supplement: Supplementary file 1 — Supplementary information [file 41598_2017_2286_MOESM1_ESM.pdf]

Daily corticosterone rhythm modulates pineal function through NFκB-related gene transcriptional program

Sanseray da Silveira Cruz-Machado; Eduardo K. Tamura; Claudia E. Carvalho-Sousa; Vanderlei Amadeu Rocha; Luciana Pinato; Pedro A. C. Fernandes; Regina P. Markus.

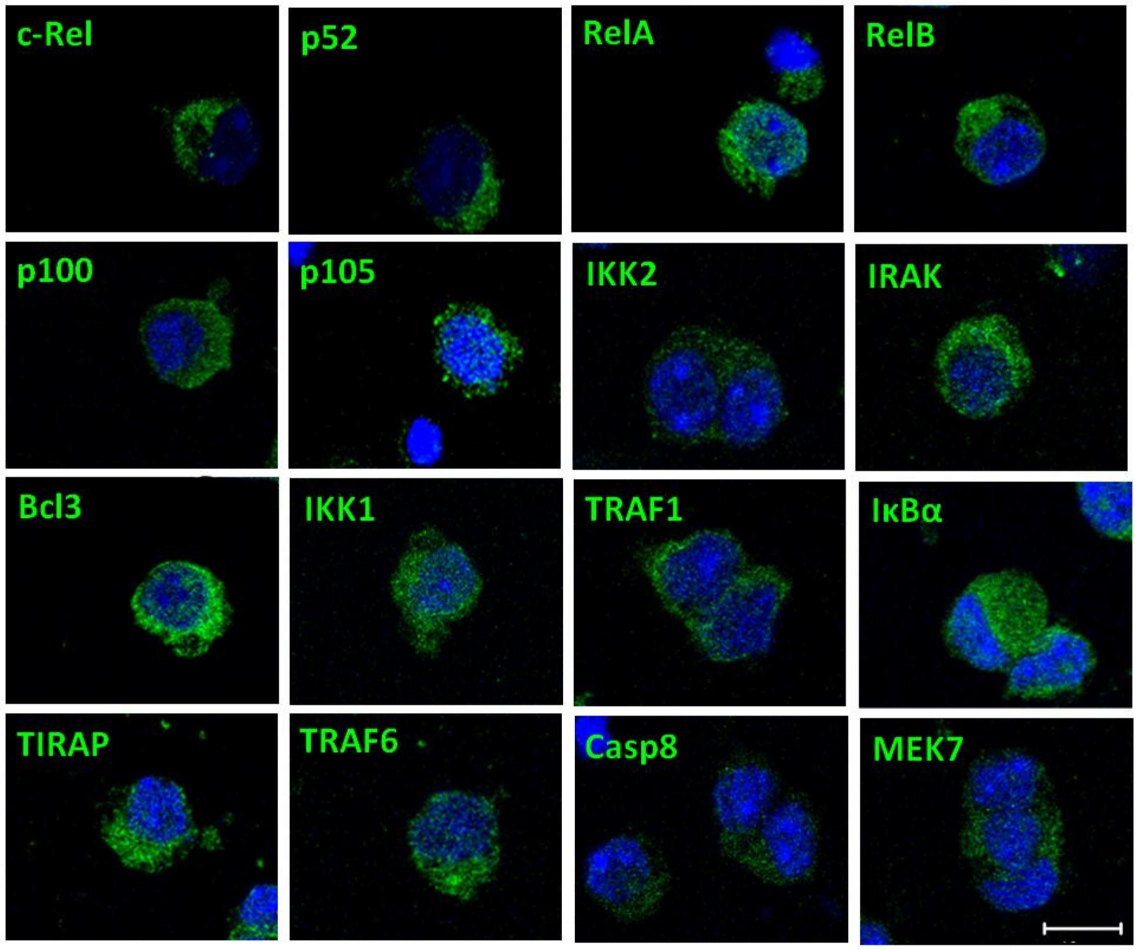

Suppl. Fig. 1 – Pinealocyte expresses 16 proteins related to NFκB signaling pathway. Cells were observed by confocal microscopy. Green staining represents the fluorescence from FITC and blue staining represents the fluorescence from DAPI, a nuclear marker. Scale bar: 10 µm.

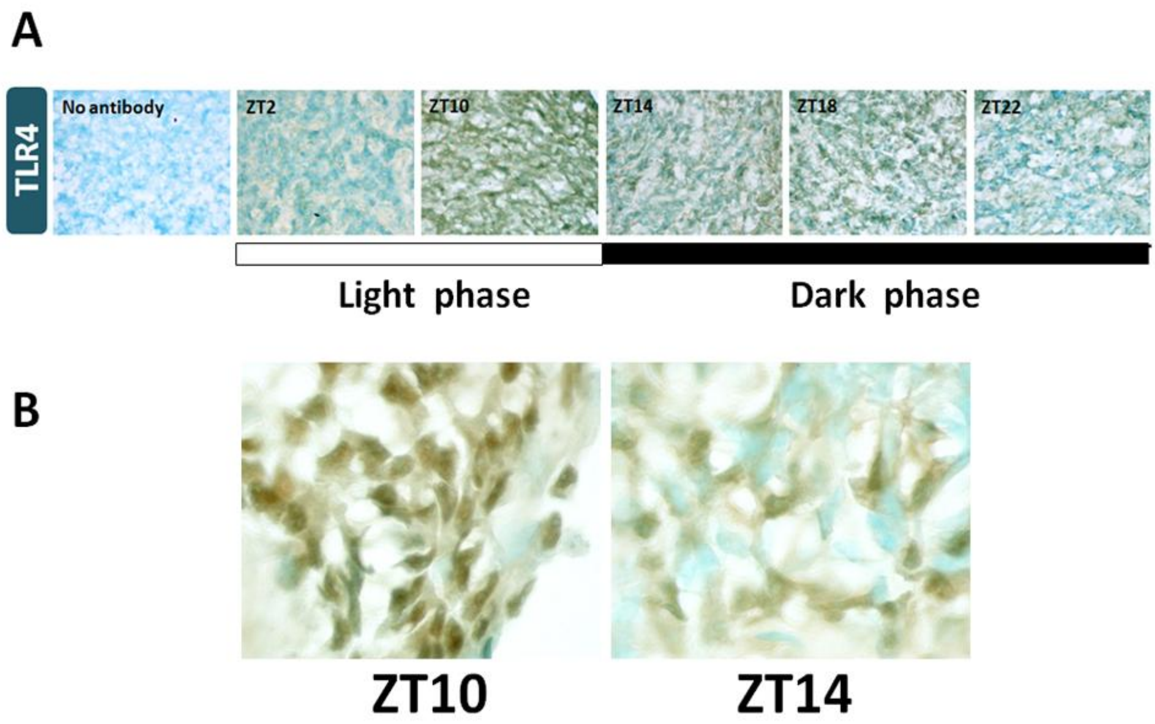

Suppl. Fig. 2 – Expression of TLR4 protein in cryosections from rat pineal glands. Bright-field photomicrograph shows part of pineal parenchyma demonstrating diffuse presence of TLR4 expression during light (ZT2, ZT10) or dark phases (ZT14, ZT18, ZT22) (20x objective). Note that stronger immunoreactivity for TLR4 occurred at ZT10 (end of light phase). B) Higher magnification of TLR4-positive cells in the rat pineal gland at ZT10 and ZT14 (100x objective). For negative controls, no primary antibody was incubated.

Supplementary table 1 – List of genes evaluated in the qPCR array profiler Rat TLR signaling pathway (PARN-018A, SABioscience, Frederick, MD, USA) including the GeneBank access number, symbols, gene names and aliases.

GenBank	Symbol	Gene name	Aliases
**NM_001007798**	*Btk*	Bruton agammaglobulinemia tyrosine kinase	-
**NM_022277**	*Casp8*	Caspase 8	-
**NM_031530**	*Ccl2*	Chemokine (C-C motif) ligand 2	MCP-1, Scya2, Sigje
**NM_021744**	*Cd14*	CD14 molecule	-
**NM_012926**	*Cd80*	Cd80 molecule	B7-1
**NM_020081**	*Cd86*	CD86 molecule	B7-2
**NM_024125**	*Cebpb*	CCAAT/enhancer binding protein (C/EBP), beta	Il6dbp, LAP, NF-IL6, TCF5
**NM_001107588**	*Chuk*	Conserved helix-loop-helix ubiquitous kinase	-
**NM_001005897**	*Clec4e*	C-type lectin domain family 4, member e	Clecsf9, Mincle
**NM_053852.1**	*Csf2*	Colony stimulating factor 2 (granulocyte-macrophage)	Gm-csf, Gmcsf
**NM_017104**	*Csf3*	Colony stimulating factor 3 (granulocyte)	-
**NM_139089**	*Cxcl10*	Chemokine (C-X-C motif) ligand 10	IP-10, Scyb10
**NM_152937**	*Fadd*	Fas (TNFRSF6)-associated via death domain	Mort1
**NM_022197**	*Fos*	FBJ osteosarcoma oncogene	c-fos
**NM_012963**	*Hmgb1*	High mobility group box 1	Ac2-008, Hmg1, MGC93598, MGC93599
**NM_001098241**	*Hras*	Harvey rat sarcoma virus oncogene	HRAS1, c-H-ras
**NM_031971**	*Hspa1a*	Heat shock 70kD protein 1A	HSP72, Hsp70-1, Hspa1, Hspa1b
**NM_022229**	*Hspd1*	Heat shock protein 1 (chaperonin)	Hsp60, Hspd1-30p
**NM_001014786**	*Ifna1*	Interferon-alpha 1	IFN-alpha1
**NM_019127**	*Ifnb1*	Interferon beta 1, fibroblast	Ifnb
**NM_138880**	*Ifng*	Interferon gamma	IFNG2
**NM_053355**	*Ikbkb*	Inhibitor of kappa light polypeptide gene enhancer in B-cells, kinase beta	AIM-1, IKK2
**NM_012854**	*Il10*	Interleukin 10	IL10X
**NM_053390**	*Il12a*	Interleukin 12a	-
**NM_017019**	*Il1a*	Interleukin 1 alpha	IL-1 alpha
**NM_031512**	*Il1b*	Interleukin 1 beta	-
**NM_013123**	*Il1r1*	Interleukin 1 receptor, type I	-
**NM_053836**	*Il2*	Interleukin 2	-
**NM_012589**	*Il6*	Interleukin 6	ILg6, Ifnb2
**NM_017020**	*Il6r*	Interleukin 6 receptor	IL6R1, Il6ra
**NM_001127555**	*Irak1*	Interleukin-1 receptor-associated kinase 1	RGD1563841
**NM_001025422**	*Irak2*	Interleukin-1 receptor-associated kinase 2	-
**NM_012591**	*Irf1*	Interferon regulatory factor 1	-
**NM_001006969**	*Irf3*	Interferon regulatory factor 3	MGC94729
**NM_021835**	*Jun*	Jun proto-oncogene	-
**NM_145095**	*Kcnh8*	Potassium voltage-gated channel, subfamily H (eag-related), member 8	ELK3, Elk1, Ets-1
**NM_001106786**	*Tbk1*	TANK-binding kinase 1	-
**NM_001097582**	*Tlr7*	Toll-like receptor 7	RGD1563357
**NM_080769**	*Lta*	Lymphotoxin alpha (TNF superfamily,	Tnfb

		member 1)	
**NM_001106405**	*Cd180*	CD180 molecule	Ly78
**NM_001024279**	*Ly96*	Lymphocyte antigen 96	MD-2
**NM_012798**	*Mal*	Mal, T-cell differentiation protein	MALGENE
**NM_001100674**	*Map2k3*	Mitogen activated protein kinase kinase 3	-
**NM_001030023**	*Map2k4*	Mitogen activated protein kinase kinase 4	MKK4
**NM_053887**	*Map3k1*	Mitogen activated protein kinase kinase kinase 1	Mekk1
**NM_001107920**	*Map3k7*	Mitogen activated protein kinase kinase kinase 7	Tak1
**NM_001106904.1**	*Map4k4*	Mitogen-activated protein kinase kinase kinase kinase 4	-
**NM_053829.1**	*Mapk8*	Mitogen-activated protein kinase 8	JNK
**NM_001100673**	*Mapk8ip3*	Mitogen-activated protein kinase 8 interacting protein 3	JIP3, JSAP1
**NM_017322**	*Mapk9*	Mitogen-activated protein kinase 9	SAPK
**NM_198130**	*Myd88*	Myeloid differentiation primary response gene 88	-
**NM_001276711.1**	*Nfkb1*	Nuclear factor of kappa light polypeptide gene enhancer in B-cells 1	NF-kB
**NM_001105720**	*Nfkbia*	Nuclear factor of kappa light polypeptide gene enhancer in B-cells inhibitor, alpha	RL, IF-1
**NM_030867**	*Nfkbib*	Nuclear factor of kappa light polypeptide gene enhancer in B-cells inhibitor, beta	MGC93398
**NM_212509**	*Nfkbil1*	Nuclear factor of kappa light polypeptide gene enhancer in B-cells inhibitor-like 1	-
**NM_001108133**	*Nfrkb*	Nuclear factor related to kappa B binding protein	-
**NM_017323**	*Nr2c2*	Nuclear receptor subfamily 2, group C, member 2	Tr4
**NM_001100565**	*Peli1*	Pellino 1	-
**NM_053373**	*Pglyrp1*	Peptidoglycan recognition protein 1	Pglyrp, Pgrp
**NM_013196**	*Ppara*	Peroxisome proliferator activated receptor alpha	PPAR
**NM_019335**	*Eif2ak2*	Eukaryotic translation initiation factor 2-alpha kinase 2	Pkr, Prkr
**NM_017232**	*Ptgs2*	Prostaglandin-endoperoxide synthase 2	COX-2, Cox2
**XM_223688**	*Rel*	V-rel reticuloendotheliosis viral oncogene homolog (avian)	-
**NM_199267**	*Rela*	V-rel reticuloendotheliosis viral oncogene homolog A (avian)	NFkB
**NM_001008349**	*Nfkb2*	Nuclear factor of kappa light polypeptide gene enhancer in B-cells 2, p49/p100	MGC93816, RGD1307189
**NM_001191865.1**	*Ripk2*	Receptor-interacting serine-threonine kinase 2	-
**NM_053588**	*Rnf138*	Ring finger protein 138	MGC72355, Rsd4, Trif
**NM_001105817**	*Sarm1*	Sterile alpha and TIR motif containing 1	-
**NM_001108890**	*Ticam2*	Toll-like receptor adaptor molecule 2	-
**NM_001172120**	*Tlr1*	Toll-like receptor 1	-
**NM_198769**	*Tlr2*	Toll-like receptor 2	-
**NM_198791**	*Tlr3*	Toll-like receptor 3	-
**NM_019178**	*Tlr4*	Toll-like receptor 4	-
**NM_001145828**	*Tlr5*	Toll-like receptor 5	-
**NM_207604**	*Tlr6*	Toll-like receptor 6	-
**NM_198131**	*Tlr9*	Toll-like receptor 9	-
**NM_012675**	*Tnf*	Tumor necrosis factor (TNF superfamily, member 2)	MGC124630, RATTNF, TNF-alpha, Tnfa
**NM_013091**	*Tnfrsf1a*	Tumor necrosis factor receptor	MGC105478, Tnfr1

		superfamily, member 1a	
**NM_001024771**	*Tnip2*	TNFAIP3 interacting protein 2	-
**NM_001109668**	*Tollip*	Toll interacting protein	-
**NM_001100480**	*Tradd*	TNFRSF1A-associated via death domain	-
**NM_001107754**	*Traf6*	Tnf receptor-associated factor 6	-
**NM_053928**	*Ube2n*	Ubiquitin-conjugating enzyme E2N (UBC13 homolog, yeast)	MGC93937
**NM_001110345.2**	*Ube2v1*	Ubiquitin-conjugating enzyme E2 variant 1	-
**NM_001007604**	*Rplp1*	Ribosomal protein, large, P1	MGC72935
**NM_012583**	*Hprt1*	Hypoxanthine phosphoribosyltransferase 1	Hgprtase, Hprt, MGC112554
**NM_173340**	*Rpl13a*	Ribosomal protein L13A	-
**NM_017025**	*Ldha*	Lactate dehydrogenase A	Ldh1
**NM_031144**	*Actb*	Actin, beta	Actx

Supplementary table 2 – List of antibodies, specification, manufacturer and dilution used for the immunofluorescence assay presented in supplemental figure 1.

Antibody	Specification	Manufacturer	Dilution
NFκB p65 (RelA)	Rabbit polyclonal	Santa Cruz Biotechnology (sc-372x)	1:100
NFκB RelB	Rabbit polyclonal	Santa Cruz Biotechnology (sc-226x)	1:200
NFκB c-Rel	Rabbit polyclonal	Santa Cruz Biotechnology (sc-70x)	1:100
NFκB p52	Rabbit polyclonal	Santa Cruz (sc-298x)	1:100
NFκB p100/p52	Rabbit polyclonal	Abcam (AB7972)	1:100
NFκB p105/p50	Rabbit polyclonal	Abcam (AB7971)	1:100
Bcl3	Rabbit polyclonal	Santa Cruz Biotechnology (sc-185x)	1:300
IKK1	Rabbit polyclonal	Santa Cruz Biotechnology (sc-7218)	1:100
IKK2	Rabbit polyclonal	Abcam (AB59195)	1:100
IRAK	Rabbit polyclonal	Abcam (AB238)	1:50
TIRAP	Rabbit polyclonal	Santa Cruz Biotechnology (sc-28822)	1:50
TRAF6	Rabbit monoclonal	Merck Milipore (04-452)	1:50
TRAF1	Rabbit polyclonal	Santa Cruz Biotechnology (sc-7186)	1:50
IκB-alpha	Rabbit polyclonal	Santa Cruz Biotechnology (sc-371)	1:100
Caspase 8	Rabbit polyclonal	Novus Biologicals (NBP1-05123)	1:50

MEK7	Rabbit monoclonal	Abcam (AB52618)	1:50
